# A single-blinded trial of methotrexate versus azathioprine as steroid-sparing agents in generalized myasthenia gravis

**DOI:** 10.1186/1471-2377-11-97

**Published:** 2011-08-05

**Authors:** Jeannine M Heckmann, Amanullah Rawoot, Kathleen Bateman, Rudi Renison, Motasim Badri

**Affiliations:** 1 Department of Medicine, Division of Neurology, University of Cape Town, Observatory, Cape Town, 7925, South Africa; 2 Department of Medicine, University of Cape Town, Observatory, Cape Town, 7925, South Africa; 3 Department of Basic Sciences, College of Medicine, King Saud bin Abdulaziz University for Health Sciences, Saudi Arabia

## Abstract

**Background:**

Long-term immunosuppression is often required in myasthenia gravis (MG). There are no published trials using methotrexate (MTX) in MG. The steroid-sparing efficacy of azathioprine (AZA) has been demonstrated after 18-months of starting therapy. However, AZA is considered expensive in Africa. We evaluated the steroid-sparing efficacy of MTX (17.5 mg weekly) compared with AZA (2.5 mg/kg daily) in subjects recently diagnosed with generalized MG by assessing their average monthly prednisone requirements.

**Methods:**

The primary outcome was the average daily prednisone requirement by month between the two groups. Prednisone was given at the lowest dose to manage MG symptoms and adjusted as required according to protocol. Single-blinded assessments were performed 3-monthly for 2-years to determine the quantitative MG score and the MG activities of daily living score in order to determine those with minimal manifestations of MG.

**Results:**

Thirty-one subjects (AZA n = 15; MTX n = 16) satisfied the inclusion criteria but only 24 were randomized. Baseline characteristics were similar. There was no difference between the AZA- and MTX-groups in respect of prednisone dosing (apart from months 10 and 12), in quantitative MG Score improvement, proportions in sustained remission, frequencies of MG relapses, or adverse reactions and/or withdrawals. The MTX-group received lower prednisone doses between month 10 (p = 0.047) and month 12 (p = 0.039). At month 12 the prednisone dose per kilogram bodyweight in the MTX-group (0.15 mg/kg) was half that of the AZA-group (0.31 mg/kg)(p = 0.019).

**Conclusions:**

This study provides evidence that in patients with generalized MG methotrexate is an effective steroid-sparing agent 10 months after treatment initiation. Our data suggests that in generalized MG methotrexate has similar efficacy and tolerability to azathioprine and may be the drug of choice in financially constrained health systems.

**Trial registration:**

SANCTR:DOH-27-0411-2436

## Background

Methotrexate (MTX) is a cost-effective immunosuppressant. Although anecdotal reports of its use in myasthenia gravis (MG) date back several decades, there are no published randomized or quasi-randomized trials [1]. Azathioprine (AZA) is considered by most to be the first-line steroid-sparing immunosuppressant in MG [2] and data show that it has significant steroid-sparing activity compared with prednisone alone after 18 months of treatment [3]. However, AZA is considered an expensive therapy in the developing world.

The incidence of MG in sub-Saharan Africa is expected to be similar to that elsewhere in the world were patients able to access appropriate health care [4]. Despite the need for cheaper MG therapies in developing countries, developed countries are investigating more costly alternatives [5]. As MTX is substantially cheaper than AZA it has the potential for wider availability in Africa. Efficacy data for MTX in MG is therefore of critical importance.

MTX is used effectively as an immunosuppressant in target-organ autoimmune disease such as Crohn's disease and psoriasis and it is considered the disease modifying agent of choice in rheumatoid arthritis (RA) [6]. Mertens et al. [7] alluded to the efficacy of MTX in generalized MG decades ago. However, many recent reviews on MG therapies either do not include MTX as an option [5,8,9] or only regard this agent as an alternative if other drugs have failed [10].

MTX is a structural analog of folic acid and exerts an anti-proliferative effect by metabolic interference with DNA synthesis. The safety and efficacy of MTX in RA is well established [6] and studies suggest that MTX may show earlier efficacy compared with AZA [11]. In RA, substantial data show that a weekly MTX dose of 17-20 mg produces an optimal efficacy/toxicity ratio [6,12,13].

The main objective of this study was to determine the efficacy of MTX as a steroid-sparing agent in subjects who had recently been diagnosed with generalized MG, and compare this with the efficacy of AZA. In MG, AZA has been shown to be an effective steroid-sparer compared to placebo after a treatment period of 18 months [3]. To achieve this we performed a 24-month parallel study with a design similar to that of Palace et al. [3].

## Methods

A randomized, single-blind study of AZA and prednisone as compared to MTX and prednisone was planned to recruit 60 patients who had recently been diagnosed with generalized MG. This sample size calculation was based on the proportion of patients with MG who were steroid-independent after receiving AZA vs. placebo for a period of 36 months (AZA-Placebo Δ = 40%) [3] and AZA vs. MTX at six months in inflammatory bowel disease (AZA-MTX Δ = 7%) [14], as well as taking into consideration anticipated feasibility and recommendations by Barohn et al. [15].

To meet the objective of comparing the steroid-sparing effectiveness between AZA and MTX, the primary outcome was the average prednisone dose required in each group to achieve and maintain minimal manifestation status (MMS) over 24 months. The clinical MG measurements were assessed by blinded assessors and used to adjust prednisone dosing per protocol, aiming for MMS as defined by the MGFA (i.e. no symptoms of limitation of functioning during everyday living even if some fatiguable weakness was noted on examination) [16]. The MG activities of daily living (MG-ADL) questionnaire was used to objectively assess functional status and subjects in MMS were required to have no MG symptoms (MG-ADL = 0) [17].

Secondary outcomes included the improvement in quantitative MG score compared to baseline, frequency of adverse events and treatment failures in each group. Treatment failures were defined as: withdrawal from the study whether due to intolerance of study medication or uncontrolled MG disease requiring alternative medications; hospitalization for MG relapse or intravenous antibiotic therapy; and death (all-cause mortality).

### Subjects

All patients had newly diagnosed (within the previous six months) generalized MG (MGFA class II, III or IV)[15]. A clinical diagnosis of MG had to be supported by at least one of the following: a positive acetylcholine receptor (AChR) antibody assay (AChR-Ab-positive), positive intra-muscular neostigmine test or > 10% decrement on 2-Hz repetitive nerve stimulation [18]. Subjects were required not to have been treated with steroid-sparing agents although they may have been prescribed prednisone prior to inclusion.

For inclusion the severity of MG was such that it resulted in functional disability with respect to the normal activities of daily living despite pyridostigmine therapy so that immunosuppression was indicated. Subjects with AChR-Ab-negative MG and thymoma-associated MG were included if they fulfilled the above criteria and thus required immunosuppressive therapy to control myasthenic disease. Exclusions comprised those with MG confined to the extraocular muscles or concomitant illness such as uncontrolled thyroid disease or additional polymyositis, or subjects with hepatitis B or HIV infections. Thymoma-MG subjects were stabilized on prednisone (± plasma exchange (P/E) or intravenous immune globulin (IVIg)) and surgery scheduled to be performed at the earliest possible time. Due to the concomitant recruitment of generalized AChR-Ab-positive MG patients for a thymectomy trial [19], all such patients opting for this study had refused thymectomy. Potentially child-bearing females were encouraged to use contraception. All patients were advised to refrain from alcohol use [20].

Patients entered after written informed consent and were randomized to either the AZA- or MTX-group using a computer-generated random number sequence. The principal investigator (PI)(JMH), patients and pharmacists were unblinded, but the outcome assessors (AR, KB and RR) remained blinded to the medication subjects were receiving. During the consent process (pre-randomization) it became evident that some subjects could not be randomized as they were unable to afford AZA. Due to limited study funding subjects received their assigned treatment from either the state pharmacy if indigent or from a private pharmacy if they had comprehensive medical insurance. However, participants with partial health insurance, such as a hospital-plan, were not eligible for free state-sponsored chronic medication and could not afford the more expensive AZA. These subjects opted out of randomization but agreed to follow study protocol taking MTX.

The study was approved by the University of Cape Town Health Science Faculty research ethics committee (HSF-067/2005), registered with the South African National Clinical Trials Registry (http://www.sanctr.gov.za) DOH-27-0411-2436, and performed in compliance with the Helsinki Declaration.

### Treatment protocol

The dose of AZA approximated 2.5-3 mg/kg daily and MTX 17.5 mg weekly. AZA was initiated at 50 mg daily for 5 days followed by an immediate dose escalation to approximately 2.5-3 mg/kg daily. AZA doses were adjusted in subjects gaining weight. MTX was started at 7.5 mg weekly and escalated weekly by 2.5 mg until a dose of 17.5 mg weekly. The MTX-group also received 5 mg folate for 5 days of every week (25 mg weekly) excluding the MTX dosing day and the day thereafter. Adverse events or toxicity were managed by reducing MTX by 2.5 mg weekly and AZA doses by 25 mg daily.

Medications were scripted by the PI. Prednisone dosing was adjusted according to protocol with the main objective to obtain MMS as recorded by the blinded assessors. Prednisone was initiated at 20 mg daily and escalated by 5 mg weekly until either a dose of 60 mg daily or 1 mg/kg was reached, or the patient reached MMS on a lower dose. Patients in whom prednisone had been initiated prior to study entry underwent a similar prednisone dose escalation from the entry-level dose. Hospitalized subjects underwent more rapid escalations as required. Prednisone tapering (scripted by the PI) was initiated at 5 mg decrements every month once MMS was reached, or if prednisone-related side effects intervened.

Pyridostigmine doses ranged between 180-360 mg daily depending on symptoms. The dose required at the final visit was noted. Vitamin D and Ca^2+ ^supplements were prescribed as is standard practice with prednisone. Patients experiencing dyspepsia were prescribed histamine-2 receptor antagonists (cimetidine) and, if symptoms persisted, gastroscoscopy was performed and a proton-pump inhibitor (omeprazole) initiated (standard care).

MG relapses were managed by increasing the dose of prednisone or, if severe (impending MG crisis), additional immune-suppression was administered such as P/E or IVIg. If an unscheduled visit was required, the PI arranged for staff unrelated to the study to assist in managing the patient.

Visits were scheduled at baseline-, and 1-, 2-, 4- and 6-months after study entry followed by 3-monthly visits for 2 years. At baseline the PI determined the duration of symptoms prior to the diagnosis of MG and before study entry. Pre-study cumulative daily prednisone dose and duration were documented. Patients underwent a full examination, laboratory screening including a complete blood and differential count, urea and electrolytes, liver transaminases, thyroid hormone, random serum glucose and hepatitis B and HIV serological status. A chest X-ray was performed followed by CT scanning of the mediastinum. Subject weight and blood pressure were measured at each visit and the following laboratory tests were monitored: blood counts, serum aspartate aminotransferase (AST), alanine aminotransferase (ALT), gamma glutamyltransferase (GGT) and random glucose. An alcohol intake questionnaire was completed at 6-monthly intervals according to a 3-point scale; 0 did not drink, 1 did some drinking (< 2 drinks per week), and 2 moderate drinking (1 drink most days) [21].

At each visit the blinded assessors performed the MG-ADL and the quantitative MG score [16]. The quantitative MG score was used as described with a minor modification related to hand grip. We anticipated a larger proportion of older subjects and, therefore, adjusted the normal values for the grip dynamometer from those expected for 50-year olds to normal ranges expected for 70-year old participants as suggested by the manufacturer (T.K.K. 5401, Takei Scientific Instruments). The categories were reduced by 66% to accommodate mild, moderate or severe and the value for left-handed grip was 20% below the right hand as recommended by Jaretzki et al. [16].

The PI reviewed medication related side effects and laboratory values. The AZA or MTX doses were adjusted if required. Laboratory toxicity was defined as white cell count ≤3.0 x10^9^/L, neutrophils ≤1.5 x10^9^/L, or platelets ≤100 x10^9^/L, or AST, ALT and GGT > 2-fold the upper limit of normal.

### Data management & Statistics

Outcomes related to prednisone dosing, quantitative MG score and MG-ADL were analyzed in as per-protocol analysis in which data measurements of those who withdrew were censored after the date on which they were no longer on study medication. However, data of all subjects were included in the denominator for proportionate outcomes such as MMS and number of failures and "responders". As MG could relapse with prednisone weaning, only those reaching sustained MMS defined as MMS until the end of the study at 24 months, was deemed relevant. The first treatment failure per patient was recorded in the proportional analysis (per 6-month period).

The quantitative QMG scores were modeled as ordinal variables and analyzed using a non-parametric test [22]. Quantitative MG score improvements from baseline (or diagnosis whichever worse if P/E or IVIg were administered after diagnosis but prior to baseline) were calculated by subtracting the value from those at subsequent study visits.

Patients were questioned at each visit regarding compliance. With non-compliance, the recorded dose reflected that which was taken if different from the prescription dose. Patients who missed an appointment were contacted telephonically and the doses taken and MG functional status (MG symptoms) were recorded.

Additional assessments included the number of episodes of worsening during the first year with worsening defined as > 20% deterioration in the quantitative MG score compared to baseline and the proportion of "responders" defined as quantitative MG score improvements of > 3.5 units from baseline [1,15].

For the statistical analysis, normally distributed data were presented as means with standard deviations (SD) and non- normally distributed data as median and interquartile ranges (IQR). The Student t-test, the Mann-Whitney test and the χ^2 ^test (or Fisher exact test) were used to compare data, as appropriate. All analyses were two-sided and a p-value < 0.05 was considered significant. Statistical analyses were done using STATISTICA 9 (Statsoft^®^).

## Results

**Figure 1:**
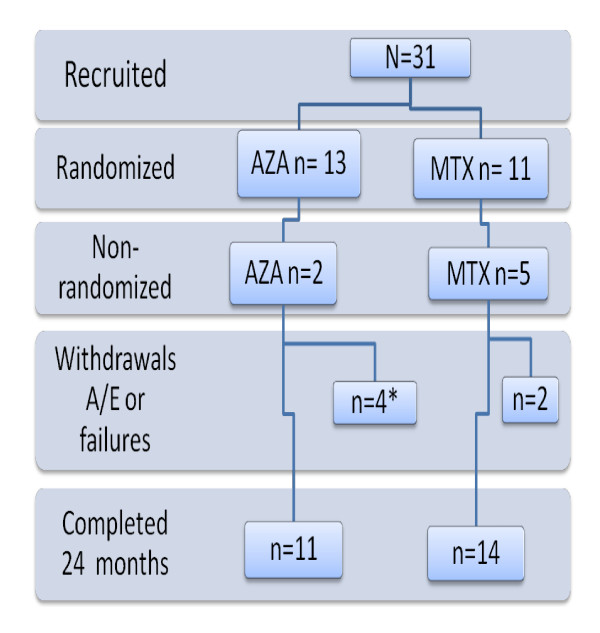
**An outline of subject participation during the study**. * 1 patient died of unrelated causes at 12.5 months (see text).

The study was conducted between 2005 and 2010 and due to slow recruitment it was halted after five years. Eight eligible subjects with AChR-Ab-positive generalized MG were either recruited to a thymectomy trial (n = 5) [19] or declined study participation (n = 3) as they lived too far from the study site. Thirty-one subjects entered the study. Twenty-four subjects were randomized to either MTX (n = 11) or AZA (n = 13). The residual 7 subjects were willing to follow study protocol but were not randomized either because they could not afford AZA if potentially randomized to that group (n = 5) and therefore included into the MTX-group, or because they opted for standard of care therapy with AZA (n = 2)(Figure 1).

The MG subtypes were similar (Table 1). Four of seven AChR-Ab- negative cases were screened for MuSK antibodies and one was positive. The symptom duration prior to a diagnosis (and treatment) of MG were similar in the two groups; two patients, one in each group, had symptom duration of almost four years. Seven participants (AZA = 3; MTX = 4) had not received any prednisone prior to study entry. The remaining subjects had received prednisone since their MG diagnosis within the previous six months, but the prednisone doses and treatment duration were similar between the two groups (Table 1). All thymoma-MG patients except two, were stabilized on prednisone (± P/E or IVIg) and surgery performed prior to study entry. One patient, who was diagnosed with thymoma on CT chest after study entry, could only undergo thymomectomy four months later and one patient persistently refused surgery throughout the study period. None of the non-thymoma MG patients underwent thymectomy (see above).

**Table 1:** Demographics and baseline characteristics of participants

	Azathioprine	Methotrexate	P value
Patients, n (Females/Males)	15 (9/6)	16 (10/6)	0.45^a^

Age (years), mean ± SD	42.7 ± 16.8	47.9 ± 14.8	0.37^b^

Weight (kg), mean ± SD	77.1 ± 21.2	76.9 ± 23.0	0.98^b^

Type of MG, n (%)			0.83^a^
AChR-Ab-positive	9 (60)	10 (62)	
Thymoma	2 (13)	3 (19)	
AChR-Ab-negative	4 (27)	3 (19)*	

Pre-study characteristics			
Symptom duration (months), mean ± SD	10.3 ± 10.6	7.5 ± 13.0	0.51^b^
Symptom duration (months), median (IQR)	6.0 (1.5; 15.0)	4.8 (2.3; 8.0)	
Patients on prednisone pre-study, n (%)	12 (80)	12 (75)	0.74^a^
Prednisone duration (months), mean ± SD	1.5 ± 1.8	1.3 ± 1.1	0.63^b^
Cumulative prednisone (mg), mean ± SD	1170 ± 1768	1032 ± 1117	0.79^b^

MGFA severity at presentation, n			
Grade 2a/2b	2/0	2/2	
Grade 3a/3b	2/3	1/5	
Grade 4a/4b/5	1/6/1	0/4/2	

Pre-study P/E or IVIg, n	1	3	

Pre-study thymomectomy, n	1 (1 delayed)	2 (1 refused)	

Concomitant disease, n			
NIDDM	1	1	
Hypertension	2	2	
Thyroid disease on stable replacement therapy	1	2	
Other autoimmune disease**	1	1	
Other disease #	3	4	

SD- standard deviation; IQR- interquartile range; AChR-Ab-positive - refers to AChR antibody positive non-thymoma MG; *1 patient MuSK-antibody positive.

NIDDM refers to non-insulin dependent diabetes mellitus. Only thymoma-MG patients underwent thymomectomies- see text.

**vitiligo & pernicious anaemia.

^# ^Other disease refers to asthma, idiopathic seizures (n = 1), peptic ulcer disease (n = 1), obesity with polycystic ovaries (n = 1), hypercholesteremia (n = 1), benign prostatic hypertrophy (n = 1); gluten sensitive enteropathy (n = 1).

^a ^χ^2 ^test. ^b ^t-test.

Baseline values between the AZA- and MTX-group were similar including the duration of MG symptoms prior to study entry or MG diagnosis (results not shown, p = 0.51) and the proportion on prednisone, as well as the cumulative prednisone exposure prior to baseline (Table 1). The baseline bodyweight, MGFA severity grade, quantitative MG scores, and proportions requiring pre-study IVIg or P/E were similar (Table 1). Seven patients (AZA = 3; MTX = 4) required hospitalization at the time of diagnosis.

**Figure 2:**
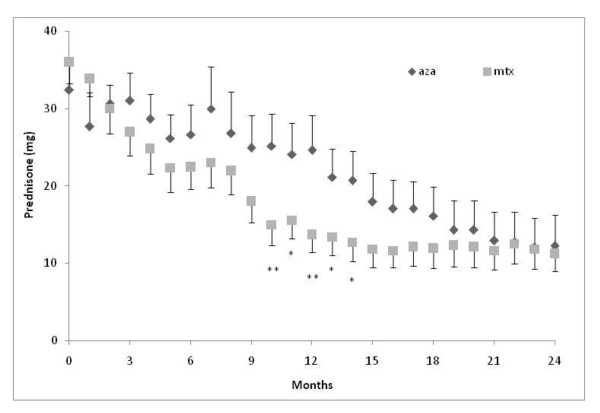
**Average daily prednisone dose by month of those remaining on their assigned steroid-sparing agent**. Prednisone dose refers to average mg per day. Error bars ± standard error of the mean. *p = 0.078; **10 months p = 0.047 and **12 months p = 0.039.

Prednisone doses were not significantly different between the AZA and MTX groups throughout the study except at months 10 (p = 0.047) and month 12 (p = 0.039) when the average daily doses by month were significantly lower (2-sided) in the MTX-group; mean difference (Δ) between MTX and AZA (MTX-AZA) at month 10 = 10.21 mg; 95% confidence interval (CI) 0.12; 20.21; mean Δ month 12 = 10.89 mg; 95% CI 0.58; 21.21. Months 11, 13 and 14 showed a trend (p = 0.078) towards lower doses of prednisone in the MTX-group (Figure 2). The average daily prednisone dose per kilogram bodyweight at month 12 was also lower in the MTX-group being on average 0.15 mg/kg prednisone (SD ± 0.08) daily as compared with that in the AZA-group of 0.31 mg/kg (SD ± 0.22)(mean Δ 0.16; 95% CI 0.29; 0.03; p = 0.019).

The quantitative MG scores over 2-years of study did not differ between the two groups and neither did the absolute change at 6-montly intervals (p = 0.40) (Table 2). Although the study examinations were scheduled at the same time and weekday throughout the study period, the timing of the preceding pyridostigmine dose in relation to each examination, was not documented. Similar proportions of subjects in each group reached sustained MMS (AZA n = 7; MTX n = 9; p = 0.83)(Table 2) with a median time to sustained MMS of 12.0 months (IQR 8.0; 16.0) in the AZA-group and 10.5 months (IQR 5.0; 16.5) in the MTX-group (p = 0.97). The proportions of treatment responders were similar in both groups (data not shown).

**Table 2:** Treatment outcomes and failures at 6-monthly intervals

	Baseline	6 months	12 months	18 months	24 months
**QMGS**					
AZA, median (IQR)	20.0 (11; 24)	7.0 (5; 8)	7.0 (5; 13)	5.0 (3; 10)	6.0 (3; 8)
MTX, median (IQR)	19.5 (16; 24)	7.5 (4; 11)	7.5 (6; 9)	8.0 (6; 10)	8.0 (6; 9)
**MG-ADL**					
AZA, median (IQR)	6.0 (3; 8)	3.0 (0; 5)	2.0 (0; 6)	1.0 (0; 1)	0 (0; 1)
MTX, median (IQR)	7.5 (4; 10)	1.0 (0; 5)	1.0 (0; 3)	0.5 (0; 1)	0 (0; 1)
MMS*					
AZA, n (%)		2/15 (13)	4/15 (27)	5/15 (33)	7/15 (53)
MTX, n (%)		3/16 (19)	5/16 (31)	5/16 (33)	9/16 (56)

**Period in months**		**0-6**	**6-12**	**12-18**	**18-24**

**Treatment failures, n**					
A/E withdrawals: AZA		2/15	2/13**	-	-
A/E withdrawals: MTX		2/16	-	-	-
MG relapses: AZA		4/15^#^	2/13^##^	-	-
MG relapses: MTX		3/16^#^	0/14	-	-

p > 0.05 for all comparisons. QMGS refers to quantitative MG score.

* MMS or minimal manifestation status refers to a) cumulative proportions in an intention to treat analysis and b) only to subjects with MMS maintained until the end of the study.

A/E refers to adverse events. ** includes 1 serious A/E: died of myocardial infarction.

^# ^2 subjects in each group received either a course of plasma exchange (P/E) or IVIg.

^## ^one subject received P/E.

Intolerable diarrhoea (AZA n = 1; MTX n = 1), hearing loss and acne (AZA n = 1) and loss of appetite (MTX n = 1) resulted in study withdrawals during the first six months (Table 2). MG relapses requiring hospitalization (with or without additional IVIg or P/E) occurred in both groups within six months and in two AZA-patients between 7-12 months. The time to first treatment failure was similar in the two groups (p = 0.99)(data not shown).

One patient (70-year old male) suffered a fatal myocardial infarction, an event judged to be unrelated to either MG or study medications (Table 2). This patient had several pre-morbid risk factors for ischemic heart disease and had been randomized to AZA. He died at 12.5 months into the study during which time his MG had improved consistently.

Adverse events recorded amongst both groups were similar apart from a preponderance of gastrointestinal events amongst the MTX-group and more anxiety and moodiness amongst the AZA-group (Table 3). The bodyweight of the subjects determined at baseline (Table 1) and subsequently at the 3-monthly visits (data not shown), were similar in the two groups (p = 0.40). No patient withdrew due to abnormal liver functions (Table 4). Elevated liver transaminases was the most frequent reason for MTX dose adjustments (3/16; 19%) to 15 mg (n = 2) and 12.5 mg weekly (n = 1). Two subjects who developed insulin-dependent diabetes did not show abnormalities of liver enzymes. Most patients reported either using no or occasional alcohol. Three subjects took moderate alcohol without any alterations in liver functions.

**Table 3:** Adverse events experienced since study entry and the action taken

System	Adverse event	AZA N = 15	MTX N = 16	Outcome
CVS	Hypertension	2	4	Resolved on antihypertensives
	Palpitations	2	0	Resolved- no action
	Pedal edema	1	2	1 -required diuretics

Digestive	Diarrhoea	2	1	2- resolve on study withdrawal 1- resolve no action
	Dyspepsia	1	7	Resolve on H2-R antagonist/PPI*
	Excessive weight gain**	7	8	↓ prednisone if accompanied by other AEs
	Gastric ulcer	0	1	Resolved on medical management
	Increased appetite	1	0	No action

Endocrine	Diabetes requiring insulin	1	1	Controlled on insulin
	NIDM new	3	1	Controlled on hypoglycemics & ↓ prednisone
	Blurred vision-lens edema	0	1	Resolve on ↓ prednisone
	Moon face	1	1	Improved on ↓ prednisone
	Mouth ulcers	0	2	Resolve- no dose change

Nervous	Anxiety, insomnia, moody	5	0	Controlled on amitryptaline & ↓ prednisone
	Cramps in legs	3	2	Controlled on K+ supplements
	Dysesthesiae in feet	0	1	Improved on ↓ prednisone
	Fatigue/asthenia	0	1	No action
	Headache	0	1	Resolve -no action
	Hearing loss	1	0	Resolve on study withdrawal
	Tremors	0	2	Intermittent; no action

Respiratory	Pneumonia	1	0	Intravenous antibiotics

Skeletal	Arthralgia	1	2	Intermittent; no action

Skin	Acne/erythema/hirsutism	1	1	Resolve on prednisone reduction/withdrawal
	Fungal infection	1	2	Resolve on topical antifungals & ↓ prednisone
	Hidradenitis suppurativa	1	0	Oral antibiotics with incision drainage

CVS refers to cardiovascular; AZA- azathioprine; MTX- methotrexate.

* H2-R antagonist (histamine-2 receptor antagonist (cimetidine n = 5) or proton pump inhibitor (omeprazole n = 2))

** weight gain during the study of more than 10% of body weight at baseline visit.

**Table 4:** Laboratory adverse events according to treatment groups, and action taken

	AZA n = 4/15	MTX N = 3/16	
Amylase > 2 × ULN	1	0	Resolved on dose reduction

AST > 2 × ULN	1	1	Resolved on dose reduction

ALT > 2 × ULN	2	1	1 resolved on dose reduction; 2 no action & stable

ALT > 3 × ULN	0	1	Improved on dose reduction- stable

GGT ≥2 × ULN	1	1	1 resolved on dose reduction; 1 no action & stable/resolved

AZA- azathioprine; MTX- methotrexate. ULN refers to upper limit of normal.

ALT refers to alanine aminotransferase, normal range 5-40 U/L, AST to aspartate amintransferase, normal 5-40 U/L, GGT to gamma-glutamyl transpeptidase, normal 0-35 U/l.

At the end of the study, incorporating dose adjustments due to adverse events (Table 4), the final average AZA dose was 2.23 mg/kg daily (SD ± 0.74; n = 11) and the final MTX dose was 16.73 mg weekly (SD ± 2.14; n = 14). The final mean pyridostigmine dose did not differ between the AZA-group (176 mg; SD ± 136) and MTX-group (150 mg; SD ± 103), p = 0.56. By the end of the study, three AZA- and four MTX-subjects had completely weaned pyridostigmine and four in each group had completely weaned prednisone.

## Discussion

We present the results of the first controlled trial using methotrexate in myasthenia gravis. In this single-blinded study involving recently diagnosed generalized MG subjects, we show that MTX has an onset of steroid-sparing efficacy from 10 to 12 months in subjects using an average dose of 17 mg weekly which is earlier than that of AZA. Two previous trials studying AZA, the comparator, found the efficacy as steroid-sparing immunosuppressant to become apparent between 15 to 18 months [1,3].

MG has a fluctuating disease course and it has been suggested that a more robust measure of treatment efficacy would be sustained minimal manifestation status (MMS) as opposed to transient MMS that may be followed by deterioration [1]. Both treatment groups in this study achieved similar proportions of participants with sustained MMS after similar treatment periods (Table 2). The responsiveness to treatment was also similar in both groups as measured by the improvement in quantitative MG score compared to baseline [15]. These outcomes are to be expected with adherence to the treatment protocol in this study as MMS was achieved by adjusting doses of prednisone in addition to the steroid-sparing effects of AZA or MTX. Steroid-sparer efficacy is regarded as being inversely related to the dose of prednisone required to maintain MMS in each group. From Table 2 it is evident that most improvement occurred in the first 6 months and was likely attributable to a steroid effect.

Longitudinal observation of MG subjects has indicated that approximately 10-15% of patients may go into spontaneous remission, usually after several years [23]. In our study the proportion of subjects in MMS (i.e. those without MG symptoms) was at least 25% in both groups at 12 months, escalating to more than 50% in both groups by 24 months (Table 2). Also, the majority of subjects had either moderate or severe disease at study entry with similar disease severity and duration in both treatment groups. It is therefore unlikely that spontaneous remissions contributed significantly to the outcomes investigated.

Study withdrawal as a result of drug intolerance was as frequent amongst those taking MTX as AZA (13%), and comparable to that observed in a previous study (11%) when these two agents were compared in patients with Crohn's disease, although only for six months [14]. A 48-week comparison between the two agents in RA found more frequent AZA withdrawals [11]. In our study, MTX was well tolerated with few patients requiring dose adjustments to manage raised transaminase levels, which did not necessarily occur in individuals with an increased body mass index as previously reported [24]. The low incidence of liver toxicity may have been due to folate supplementation [25,26]. In this study 25 mg of folate was supplemented every week although previous reports show (a) no differences between 5 mg folate weekly and higher doses in reducing MTX-toxicity and (b) that higher doses of folate may result in an individual requiring higher MTX doses to achieve efficacy [25-27]. Although we advised against consuming more than one alcoholic drink per week, others have allowed moderate alcohol intake without this apparently influencing transaminase levels in their subjects [25].

MTX has anti-inflammatory, anti-proliferative and immune-modulatory activities some of which are due to the cellular induction of adenosine and consequent suppression of NF-_K_B activation, a key transcription factor (reviewed in [28]). These effects may explain the early (within 3-6 weeks) radiographic improvements observed in MTX-treated RA subjects [12]. MTX has also been shown to have immunosuppressive effects including apoptosis and clonal deletion of activated T cells, an inhibitory effect on proliferating cells [27] including B cells, a reduction in pro-inflammatory cytokine production by T cells and macrophages [29] and CD95-dependant apoptosis of activated memory T cells [27]. In RA, accumulated data suggest that strategies to use MTX earlier with faster dose escalations and intensive regimens of 17-20 mg/week showed control in disease activity within three to four months (reviewed in [6,30]). The optimal initiation dosing strategy recommends starting at 15 mg weekly unless the patient is elderly or bodyweight is below 50 kg in which case some would start at 7.5 mg weekly [6,21].

A major limitation of our study is its small size with only 24 of 31 participants undergoing randomization. As we recruited substantially fewer subjects than we anticipated, the possibility exists that we have falsely accepted MTX treatment as equivalent to AZA when it is inferior (type 1 error). Nevertheless, our results do suggest that MTX shows an earlier onset of steroid-sparing efficacy than AZA. This study represents the "real-life" situation in that a spectrum of patients with generalized MG were recruited including AChR-Ab-negative and thymoma-associated variants: two less prevalent MG subgroups often associated with severe disease requiring immunosuppression [7,9,10,31,32]. Another limitation of our study is that we did not systematically examine MuSK antibody status of all our AChR-Ab-negative subjects. MuSK-positive MG can be difficult to manage with immunesuppressants [10]. We did identify one MuSK-positive subject who was randomized to MTX, required and responded to plasma exchange at study entry, and maintained a "responder" status throughout the 2-year of study.

Due to a concurrent study assessing the role of thymectomy in MG [19] in which AChR-Ab-positive MG subjects amenable to possible thymectomy were recruited to that study, all the patients recruited into this study had refused thymectomy and consequently this was not performed. In this manner bias might have been introduced. However, < 20% of this study population had mild generalized (MGFA Grade 2) disease. Another source of potential bias was the fact that socioeconomic status prevented five subjects from being randomly assigned to either treatment group. Other limitations, due to limited funding, included patients being unblinded to the study medications and prednisone dosing being captured by intended script dosing and verified by patient recall at the following visit. The latter may have resulted in minor inaccuracies in daily prednisone dosing.

## Conclusions

In a study population that is representative of that seen in a MG clinic, we present class III evidence [33] that an average methotrexate dose of 17 mg weekly is an effective steroid-sparer in generalized MG evident from 10-months of treatment initiation. Our data suggests that in addition to an earlier onset of action, methotrexate has similar efficacy and tolerability to azathioprine in recently diagnosed generalized MG and may be the drug of choice in financially constrained health systems.

## Competing interests

The authors declare that they have no competing interests.

## Authors' contributions

JMH conceived and designed the study, collected the data, participated in statistical analyses and data interpretation and was primary author of the manuscript. AR, KB and RR all collected data and participated in the drafting of the manuscript. MB participated in statistical analyses and reviewed final data interpretation and participated in the drafting of the manuscript. All authors read and approved the final manuscript.

## Pre-publication history

The pre-publication history for this paper can be accessed here:

http://www.biomedcentral.com/1471-2377/11/97/prepub
